# Does peripheral arterial occlusive disease influence muscle strength and exercise capacity in COPD patients?

**DOI:** 10.1590/1677-5449.004417

**Published:** 2017

**Authors:** Natacha Angélica da Fonseca Miranda, Cássia da Luz Goulart, Audrey Borghi e Silva, Dannuey Machado Cardoso, Dulciane Nunes Paiva, Renata Trimer, Andréa Lúcia Gonçalves da Silva

**Affiliations:** 1 Universidade de Santa Cruz do Sul – UNISC, Santa Cruz, RS, Brazil.; 2 Universidade Federal de São Carlos – UFSCar, São Carlos, SP, Brazil.; 3 Universidade Federal do Rio Grande do Sul – UFRGS, Porto Alegre, RS, Brazil.

**Keywords:** COPD, exercise test, exercise tolerance, peripheral arterial disease, DPOC, teste de esforço, tolerância ao exercício, doença arterial periférica

## Abstract

**Background:**

The pathophysiology of chronic obstructive pulmonary disease (COPD) is complex and understanding of it has been changing in recent years, with regard to its multisystemic manifestations, especially peripheral dysfunction and its influence on intolerance to exercise.

**Objectives:**

To evaluate the relationship between peripheral arterial occlusive disease (PAOD) and peripheral muscle strength and exercise capacity in COPD patients.

**Methods:**

We conducted a cross-sectional study of 35 patients with COPD who were evaluated with the Ankle-Brachial Index, handgrip strength test, 1 repetition maximum (1RM) of knee extensors and flexors, and distance covered in the incremental shuttle walking test (dISWT).

**Results:**

COPD patients with coexisting PAOD had lower dominant handgrip strength test results (33.00 vs. 26.66 kgf, p = 0.02) and worse performance in the dISWT (297.32 vs. 219.41 m, p = 0.02) when compared to the COPD patients without PAOD. Strong correlations were found between the result of the handgrip strength test and both the dISWT (r = 0.78; p < 0.001) and the 1RM/knee extension (r = 0.71; p = 0.03); and also between the dISWT and both the 1RM/knee extension (r = 0.72; p = 0.02) and the 1RM/knee flexion (r = 0.92; p < 0.001). The linear regression model showed that the dISWT variable alone explains 15.3% of the Ankle-Brachial Index result (p = 0.01).

**Conclusion:**

COPD patients with PAOD exhibit reduced muscle strength and lower exercise capacity than COPD patients without PAOD.

## Resumo

**Contexto:**

A doença pulmonar obstrutiva crônica (DPOC) apresenta uma complexa fisiopatologia e sua compreensão vem se modificando nos últimos anos, com atenção para as manifestações multissistêmicas, em especial a disfunção periférica e sua influência na intolerância ao exercício físico.

**Objetivo:**

Avaliar o impacto da doença arterial periférica obstrutiva (DAOP) na força muscular periférica e na capacidade de exercício dos portadores de DPOC.

**Métodos:**

Estudo transversal realizado com 35 portadores de DPOC, que foram avaliados pelo índice tornozelo-braquial, teste de força de preensão palmar (FPP), uma repetição máxima (1RM) de extensores e flexores de joelho, e a distância no *incremental shuttle walking test* (dISWT).

**Resultados:**

Portadores de DPOC com DAP coexistente apresentaram menor FPP da mão dominante (33,00 *versus* 26,66 kgf, p = 0,02) e pior desempenho no dISWT (297,32 *versus* 219,41 m, p = 0,02) quando comparados aos portadores de DPOC sem DAP. Fortes correlações foram encontradas entre a medida da FPP e a dISWT (r = 0,78; p < 0,001) e a 1RM/extensão de joelho (r = 0,71; p = 0,03); entre a dISWT e a 1RM/extensão de joelho (r = 0,72; p = 0,02) e a 1RM/flexão de joelho (r = 0,92; p < 0,001). O modelo de regressão linear identificou que a variável dISWT explica isoladamente 15,3% do resultado do índice tornozelo braquial (p = 0,01).

**Conclusão:**

Portadores de DPOC com DAOP coexistente apresentam maior perda da força muscular periférica e pior desempenho da capacidade de exercício quando comparados aos portadores de DPOC sem DAOP.

## INTRODUCTION

Chronic obstructive pulmonary disease (COPD) is characterized by airflow limitation, its progressive nature has been associated with an enhanced chronic inflammatory response to inhaled particles and noxious gases, and smoking is the major risk factor.^1^ Understanding of the pathophysiology of COPD has been changing in recent years, in response to its complex nature, the presence of comorbidities, and its multisystemic manifestations.^2^ Chronic systemic inflammation provokes reductions in exercise capacity, muscle strength and endurance, and quality of life.^1^^,^ 3 Peripheral muscle dysfunction is one of the most important extrapulmonary manifestations in the pathophysiology of COPD and it has been described as presence of decreased tropism and/or muscle atrophy, loss of strength and early muscle fatigue in response to exercise.^4^^,^ 5

Along the same lines, current studies report that peripheral arterial changes are present in COPD patients, with coexistence of common risk factors (e.g., smoking and chronic inflammation), and this can change the blood flow and affect cardiovascular hemodynamics.^6^^-^ 8 Peripheral arterial occlusive disease (PAOD) results from an atherosclerotic process affecting the coronary arteries, provoking occlusion of arteries in the lower limbs and can be present in COPD patients, although sometimes it may be asymptomatic and so its prevalence will be underestimated.^9^ This is widely variable around the world^6^ and in Brazil there is still a lack of records in the current literature. Identification is crucial, since it increases the risk of a cardiovascular event, increasing morbidity and mortality of patients with COPD.^7^^,^ 10

It is possible to diagnose PAOD while still in the asymptomatic stage by analyzing the Ankle-Brachial Index (ABI), which is a simple and noninvasive method.^7^ Although underused in clinical practice, the reproducibility of this test is related to calculating ABI values, for which scores of < 0.9 are defined as indicating PAOD and ABI ≥ 0.9 as absence of PAOD.^9^ Studies of the influence of PAOD on physical exercise with constant workload have been conducted, but the results are controversial.^6^^,^ 11 ^,^ 12 Furthermore, it is not known if asymptomatic PAOD is associated with worse performance by COPD patients during exercise with incremental loads (e.g., the incremental shuttle walking test [ISWT]), recruiting physiological responses from the cardiorespiratory system that reflect aerobic capacity, making it safer for adminsitration to patients with respiratory diseases.^2^ Our study aimed to evaluate the relationship between PAOD and peripheral muscle strength and exercise capacity in COPD patients. A secondary objective was to predict the ABI from ISWT. We hypothesized that the presence of the PAOD, even asymptomatic, compounded by reduced peripheral muscle strength in COPD patients, negatively affects their performance in the ISWT and impacts on the distance covered.

## METHODS

### Study design

We conducted a cross-sectional study using a non-probabilistic convenience sample at a Pulmonary Rehabilitation Program (PRP) in a University Hospital, in which all patients were recruited from the same PRP. After subjects had attended an interview, they underwent anthropometric evaluation, spirometry assessment, peripheral muscle strength testing, and measurement of ABI and exercise capacity. This research project was duly approved by the Ethics Committee (protocol number 970.251/2015) and all the participants involved agreed and signed free and informed consent forms.

### Subjects

Patients who had a clinical and functional diagnosis of COPD were selected and screened against the inclusion and exclusion criteria for the study and were then allocated to the normal ABI or PAOD groups. The study recruited 35 clinically stable and sedentary COPD patients. COPD patients with musculoskeletal disorders and/or neurologic disorders affecting the musculoskeletal system, cognitive deficit, skin lesions in the foot area, exacerbation of COPD within 30 days prior to the study, or uncontrolled ischemic heart disease were excluded from the study.

### Data collection

All patients answered an international questionnaire to collect demographic data, smoking status and clinical history. Body weight, height, and blood pressure were measured and body mass index (BMI) was calculated. The Borg rating of perceived exertion for lower limbs, Borg dyspnea score, intermittent claudication,^13^ and smoking status were also evaluated using a self-report questionnaire developed by Fagerstrom.^14^

### Pulmonary function

Pulmonary function was evaluated using digital spirometry (EasyOne^®^, Model 2001, Zürich, Switzerland) to obtain the variables forced expiratory volume in one second (FEV_1_) and the ratio FEV_1_/forced vital capacity (FVC). Spirometry was performed according to recommendations from the American Thoracic Society^15^ and the results analyzed according to predicted values.^16^ Severity of COPD airflow limitation was classified according to GOLD criteria as moderate (GOLD II), severe (GOLD III), or very severe (GOLD IV).^1^

### Peripheral muscle dysfunction

Peripheral muscle dysfunction was measured using hand grip strength (HGS) and 1-repetition maximum (1RM). The HGS was conducted with a Jamar® dynamometer (California, USA) which recommends that the subject perform the test seated, with the dominant shoulder adducted, elbow in flexion of 90°, the forearm in neutral position and the fist in a position that can vary from 0º to 30° of extension. Grip was measured three times and the average was taken.^17^ The 1RM test has shown itself to be clinically reproducible, applied to the muscle groups involved in knee flexion and extension. The 1RM strength test was used to determine the greatest amount of weight that the patient could move in a single repetition, with a random initial load that was increased or reduced in accordance with the individual’s ability to perform a repetition; this could be repeated again, with a 1-minute interval between each load.^18^ The load used during the test was increased whenever the patient could overcome the initial load with ease.

### Measurement of ABI

ABI was measured using a non-invasive vascular screening device (DV –2001, MEDPEJ^®^, Brazil) and sphygmomanometer (Welch Allyn^®^, United States of America). Systolic Blood Pressure (SBP) was measured in the brachial artery of both arms and in the posterior tibial and dorsal pedal arteries of both legs. The ABI for each leg was defined as the ratio between the highest lower limb SBP value and the highest upper limb SBP value. Patients with ABI scores of < 0.9 were defined as having PAOD, and those with ABI scores of ≥ 0.9 as not having PAOD.^19^ It has high sensitivity (79 to 95%) and specificity (95 to 96%) in comparison to the gold standard angiography.^11^

### Functional capacity by incremental shuttle walking test

The ISWT test was performed as described by Holland et al. in a 10-meter corridor, marked at each end by a cone with an inset of 0.5 meters.^20^ The subject was informed of the incremental nature of the test, which is indicated by pre-recorded sound media consisting of short and long signals. The short signals demarcate the pace of gait during each level and the long signal indicates an increase in the cadence of walking. The test is terminated when the subject exhibits intolerance or when they do not reach the end of the corridor before the two beeps in succession.^21^^,^ 22 If peripheral oxygen saturation fell below (SpO_2_) ˂ 86%, oxygen supplementation was supplied.^23^ The Incremental Shuttle Walking Test (ISWT) was developed for patients with COPD as a maximum test because it provokes physiological changes, similar to the test of cardiopulmonary exercise performed on a treadmill or cycloergometer.^20^

### Statistical analysis

Data were analyzed using the statistical analysis software SPSS^®^ (version 20.0). Results were tested for normality using the Shapiro-Wilk test and presented descriptively as means with standard deviations ($\bar{x}$ ± sd), frequencies (%) and medians (minimum and maximum). Student’s *t* test was used to reject the null hypothesis for parametric variables and the Mann-Whitney test was used for nonparametric variables. In COPD patients with coexisting PAOD, the association between variables was evaluated using the Spearman correlation. A simple linear regression model was used to evaluate the possibility of predicting ABI from ISWT. The significance of the final model was estimated with the ANOVA F test and the quality of fit by calculating the coefficient of determination (adjusted R^2^). Residuals were evaluated according to the assumptions of normality, constant variance and independence. Results with p < 0.05 were considered significant.

## RESULTS

The COPD patients evaluated were stratified into two groups according to their ABI results: Normal ABI group or PAOD group. Table 1 lists the clinical characteristics of these patients and their test results.

**Table 1:** COPD patients’ clinical characteristics and performance in tests.

**Variables**	**COPD (n = 19)** **Normal ABI**	**COPD (n = 16)** **PAOD**	**p-value**
Sex, male n (%)	13 (68.40)	8 (50.00)	0.26
Age (years)	62.84±5.69	65.69±8.70	0.25
Ethnicity			
Caucasian n (%)	16 (84.20)	15 (93.80)	0.37
Non-caucasian n (%)	3 (15.80)	1 (6.30)	0.37
BMI (kg/m^2^)	28.1±6.52	26.79±6.64	0.47
BMI classification			
Underweight n (%)	2 (10.50)	3 (18.80)	0.41
Normal weight n (%)	6 (31.60)	6 (37.50)	0.49
Obesity n (%)	11 (57.90)	7 (43.80)	0.31
FEV_1_ (L/s)	1.15±0.49	0.88±0.38	0.08
FEV_1%_ predicted	42.12±16.94	37.64±13.77	0.40
FEV_1_/FVC % predicted	65.14±18.11	62.06±19.10	0.63
GOLD Classification*			
II n (%)	5 (26.30)	3 (18.80)	0.45
III n (%)	8 (42.10)	9 (56.20)	0.31
IV n (%)	6 (31.60)	4 (25.00)	0.48
Smoking Status			
Current n (%)	4 (21.1)	2 (12.4)	0.41
Former n (%)	15 (78.9)	14 (87.6)	0.70
Smoking cigarettes/Years	6838.9±3248.3	7710.6±2831.7	0.41
Claudication Presence			
Yes n (%)	5 (26.30)	3 (18.80)	0.59
Perceived Exertion-Borg Scale	11.47±2.89	12.31±2.72	0.38
Modified-Borg Scale Dyspnea Index	3 (1-7)	3 (0-8)	0.81
ABI	1.02±0.06	0.82±0.09	≤0.01
HandGrip (kgf)	33.00 (16.00-52.66)	26.66 (11.66-32.33)	0.02
HandGrip. % predicted	101.76 (49.47-172.53)	86.46 (41.06-140.15)	0.09
dISWT (meters)	297.32±74.62	219.41±117.18	0.02
dISWT % predicted	41.98±13.55	33.76±16.36	0.11
1RM			
Knee flexion (kg)	4.08 (1.27-9.24)	2.40 (1.13-12.70)	0.39
Knee extension (kg)	7.92 (1.23-10.20)	2.72 (1.23-13.60)	0.44

Data are presented as mean ± SD or median (minimum and maximum).

n (%), number sample (frequency); ABI: ankle-brachial index; BMI: body mass index; dISWT: distance incremental shuttle walking test; FVC: forced vital capacity; FEV_1_: forced expiratory volume in 1 second; FEV1/FVC: forced expiratory volume in the first second and forced vital capacity; GOLD: Global Initiative for Chronic Lung Disease^1^; PAOD: peripheral arterial disease. The Student’s *t*-test was used when data were normally distributed; otherwise, the nonparametric Mann-Whitney test was used.

**Figure 1:**
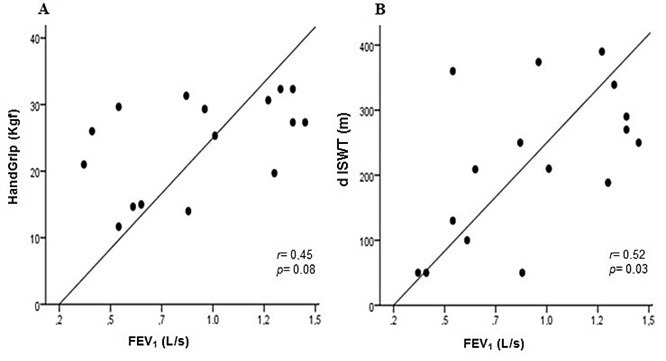
Relationship between airway obstruction and functional capacity. **(A)** Correlation between HandGrip and FEV_1_. •COPD with coexisting PAOD (n = 16). FEV_1_: forced expiratory volume in 1 second. **(B)** Correlation between dISWT and FEV_1_. dISWT: distance in incremental shuttle walking test; The association between the variables was analyzed using Spearman´s correlation test.

**Figure 2:**
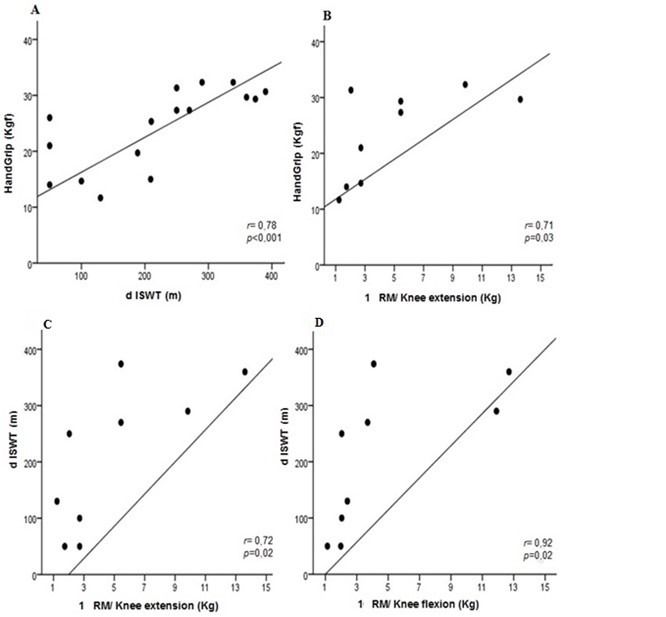
Relationship between peripheral muscle strength and functional capacity. **(A)** Correlation between HandGrip and dISWT. •COPD with coexisting PAOD (n = 16). dISWT, distance in incremental shuttle walking test. **(B)** Correlation between HandGrip and 1 RM/Knee extension. •COPD with coexisting PAOD (n = 9). RM: Repetition maximum. **(C)** Correlation between dISWT and 1 RM/ Knee extension. •COPD with coexisting PAOD (n = 9). **(D)** Correlation between dISWT and 1 RM/ Knee flexion. •COPD with coexisting PAOD (n = 9). dISWT, distance in incremental shuttle walking test; RM, repetition maximum. The association between the variables was analyzed using Spearman´s correlation test.

With the exception of dominant HGS (kgf) and performance in the ISWT (m), the clinical characteristics of the two groups were equivalent. The COPD patients with PAOD had lower HGS and worse dISWT than the COPD patients with normal ABI. In the PAOD group, there were correlations between FEV_1_ and HGS (r = 0.49; p = 0.05) and between FEV_1_ and dISWT (r = 0.56; p = 0.02) (Figure 1). Hand grip strength was strongly correlated with dISWT (r = 0.78; p < 0.001) and with 1RM/knee extension (r = 0.71; p = 0.03) (Figure 2). The results for dISWT were also strongly correlated with 1RM/knee extension (r = 0.72; p = 0.02) and with 1RM/knee flexion (r = 0.92, p < 0.001) (Figure 2). These results confirm that COPD patients with coexisting PAOD exhibited worse performance in the ISWT.

We applied a regression model and we found that dISWT alone explains 15.3% of the ABI result (p = 0.012) and so we propose an equation for prediction of ABI from dISTW: (0.783 + 0.001x dISTW) (Table 2).

**Table 2:** Simple linear regression analysis to predict ABI from distance-ISWT.

**Variables**	**Coefficient-beta**	**p-value**	**95%CI**
Constant	0.783	< 0.001	0.651-0.916
Diswt	0.001	0.012	0.000-0.001

95%CI: 95% confidence interval; ABI: ankle–brachial index; dISWT: distance in incremental shuttle walking test.

R^2^_adjusted_ = 0.153; F = 7.15 (p = 0,012). Predictive equation for ABI: (0.783+0.001x dISTW).

## DISCUSSION

Our study found that COPD patients with PAOD had lower HGS and worse dISWT than COPD patients with normal ABI. Furthermore, we observed a strong relationship between reduction in HGS and distance covered in the ISWT in COPD patients with coexisting PAOD.

Patients in the early stage of COPD exhibit peripheral skeletal muscle mass loss, which increases in the advanced stage of the disease.^24^ The decrease in muscle strength and endurance is because of changes in the composition and proportion of muscle fibers, resulting from chronic oxidative stress, which triggers early use of anaerobic metabolism, inducing limb fatigue and low exercise tolerance.^24^^,^ 25

Another important factor is that the changes in peripheral muscle strength do not affect the muscle groups uniformly, and it is known that lower limb strength is reduced more than upper limb strength.^26^ Proximal upper limb muscle strength is more affected than distal upper limb muscle strength, because it is used in activities of daily life. In COPD patients with moderate to severe disease subjected to a fatigue protocol, comparison of reduced upper limb muscle strength (elbow flexors) with reduced lower limb muscle strength (knee extensors) revealed increased perception manifest as higher fatigue scale scores (for elbow flexors and knee extensors) before and after the protocol.^27^ However, the fatigability of elbow flexors was greater than that of knee extensors.^26^ Because of continuing controversy over distribution of muscle dysfunction between upper and lower limbs in patients with COPD and because activities that involve shoulder girdle and upper limb muscles lead to a marked sensation of dyspnea, our study contributes to and expands the discussion on the relationship between hand grip relative and incremental load tests, where arm balance is fundamental to maintenance of walking cadence and performance during the test.

Regarding the ability to sustain physical activity, this is intrinsically linked to oxygen transport pathways, which depend on interaction between multiple organs, such as heart and blood vessels, lungs, respiratory and peripheral muscles, and autonomic and voluntary nervous system.^28^ Research into dysfunction of tissue oxygen transport due to altered diffusion or perfusion mechanisms is needed to improve understanding of the pathophysiological abnormalities shared by common diseases such as COPD and heart failure. It is important to highlight that there is growing evidence in the literature on the mechanisms involved in reduction of blood flow and oxygenation, both in the brain and in peripheral muscles during exercise, with particular emphasis on hypoxemia, which is common in patients with COPD, and chronic inflammation that leads to oxidative stress and endothelial dysfunction in older age.^9^^,^ 28 ^,^ 29

Patients with COPD have endothelial dysfunction that can be manifest with loss of the capacity to dilate the brachial artery, probably due to lung hyperinflation and distention of the rib cage,^9^ and arterial stiffness.^8^ However, little is known about COPD with coexisting asymptomatic PAOD and their influence on exercise capacity and on limb muscle strength. To date, only one other study had investigated the presence of asymptomatic PAOD using the ABI method and its relation with the endurance walking test in COPD patients and, in contrast with our study, they did not detect an association between the variables.^6^ The ISWT induces progressive effort and recruitment of physiological responses from the cardiorespiratory system that reflect aerobic capacity and has been administered to subjects participating in cardiac rehabilitation programs for exercise prescription, to evaluate the effectiveness of rehabilitation programs (heart and lungs), and to determine disease prognosis.^30^^,^ 31 For patients with moderate and severe COPD, the ISWT can be used an alternative to tests hitherto adminstered in laboratories and it can also be used for prescription of walking exercise.^30^ The ISWT is considered less stressful to the cardiovascular system than the treadmill test because of the progressive load imposed and is therefore safer for use with patients with respiratory diseases.^32^

Thus, based on the results of our study, we propose an equation for predicting ABI from dISTW: (0.783 + 0.001x dISTW). The ISTW is a simple, rapid, non-invasive test that is easy to administer because it does not require technology. The participants in our study tolerated the test well, despite the fact that they are seniors who have some difficulty with locomotion and with maintaining walk cadence, and the factors that prevented them from finishing the test were mostly dyspnea and fatigue in the legs.

However, our study has some limitations that should be considered, such as the 1 RM evaluation method, which does not allow us additional inferences about precise parameters of muscle strength, such as isokinetic dynamometry offers. Although the results were significant, we suggest conducting a larger cohort study to validate the equation for prediction of ABI from ISTW. Another limitation of the study is that some patients with normal ABI presented claudication, which could be of non-vascular origin, but we did not evaluate other possible causes.

In conclusion, presence of PAOD in COPD patients has a negative impact on peripheral muscle strength and the capacity to sustain a progressive exercise load compared to COPD patients without PAOD. As future prospects, further tests are needed to assess systemic pro-inflammatory factors and the effectiveness of pulmonary rehabilitation programs for improvement of peripheral muscle and arterial dysfunction in COPD patients with coexisting PAOD.

### Confidentiality of data

The authors declare that they have complied with their institution’s protocols for publication of patient data and that all patients included in the study were given sufficient information and provided written informed consent to participation in the study.

### Right to privacy and informed consent

The authors have obtained written informed consent from the patients or subjects mentioned in the article. The corresponding author is in possession of this document.
